# Spatiotemporal control of kinases and the biomolecular tools to trace activity

**DOI:** 10.1016/j.jbc.2024.107846

**Published:** 2024-10-01

**Authors:** Jeremy C. Burton, Fredejah Royer, Neil J. Grimsey

**Affiliations:** Department of Pharmaceutical and Biomedical Sciences, College of Pharmacy, University of Georgia Athens, Athens, Georgia, USA

**Keywords:** kinases, spatial signaling, FRET, p38, GPCR, atypical-p38

## Abstract

The delicate balance of cell physiology is implicitly tied to the expression and activation of proteins. Post-translational modifications offer a tool to dynamically switch protein activity on and off to orchestrate a wide range of protein-protein interactions to tune signal transduction during cellular homeostasis and pathological responses. There is a growing acknowledgment that subcellular locations of kinases define the spatial network of potential scaffolds, adaptors, and substrates. These highly ordered and localized biomolecular microdomains confer a spatially distinct bias in the outcomes of kinase activity. Furthermore, they may hold essential clues to the underlying mechanisms that promote disease. Developing tools to dissect the spatiotemporal activation of kinases is critical to reveal these mechanisms and promote the development of spatially targeted kinase inhibitors. Here, we discuss the spatial regulation of kinases, the tools used to detect their activity, and their potential impact on human health.

The packing and regulation of proteins in cells requires orchestrating their location and interaction with adaptors, cofactors, and substrates. The spatial organization of core biochemical processes, including signal transduction, is essential for cellular homeostasis and health. Central to this is the compartmentalization of signaling machinery by association with or encapsulation by membranes, organelles, and membraneless condensates. This concept of signaling compartmentalization is also utilized by receptor-mediated signaling cascades such as receptor tyrosine kinase (RTK) and G-protein receptor kinases (GPCRs). Compartmentalization of the downstream host factors, which are associated with receptor activation, plays a role in a variety of cellular functions (1, 2, 3). The discrete environmental conditions and substrate concentrations encoded within each location mediate signaling specificity for rapid and selective responses during development and stress, both homeostatic and pathological.

In addition to location, post-translational modifications (PTMs) of proteins govern their specific protein-protein interactions (PPIs), activity (activation and inhibition), secretion, and lifespan before degradation and recycling. These modifications include ubiquitylation, palmitoylation, acetylation, and phosphorylation. For functional roles and recent reviews, see (Fig. 1). For the rest of this manuscript, we will focus on arguably one of the most critical PTMs: protein phosphorylation.

**Figure 1:**
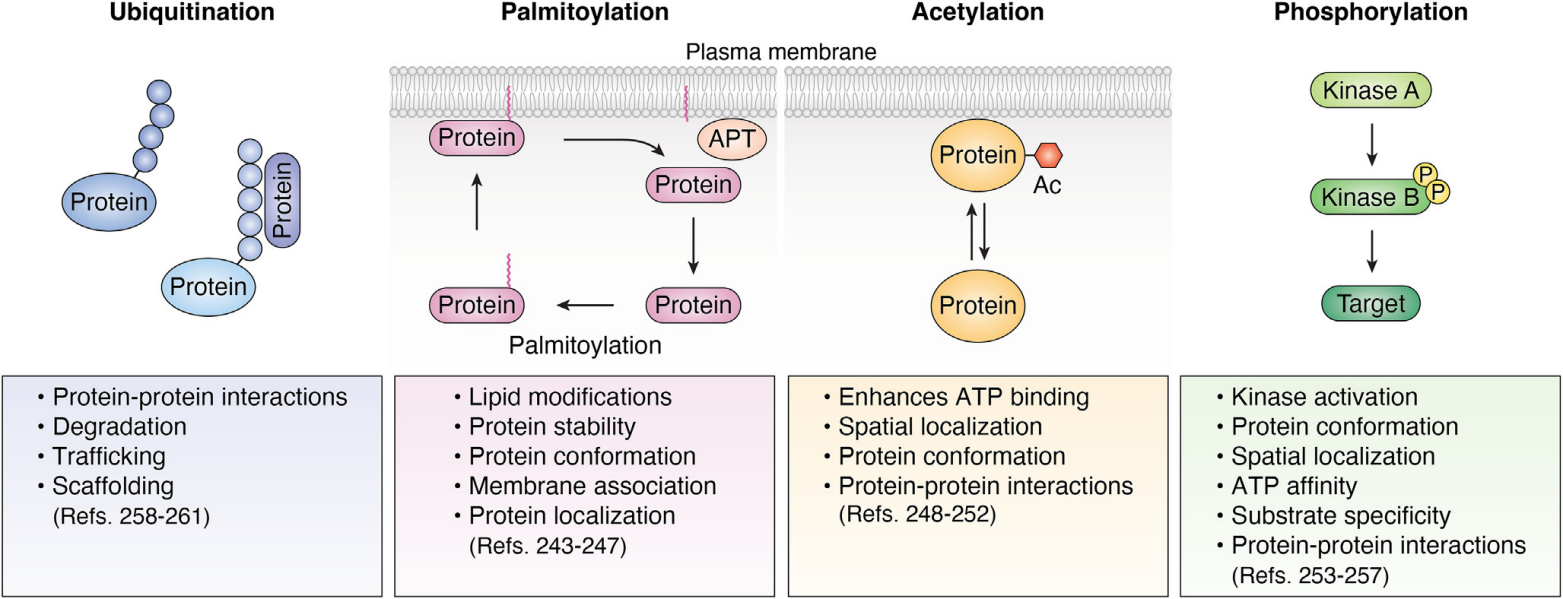
**Post-translational modifications (PTMs) and their role in cellular physiology.** Proteins can be dynamically regulated by modifications, including ubiquitination, palmitoylation, acetylation, and phosphorylation. Each PTM changes the protein, altering its functional responses. Palmytoylation (240, 241, 242, 243, 244), acetylation (245, 246, 247, 248, 249), phosphorylation (250, 251, 252, 253, 254), and ubiquitination (255, 256, 257, 258, 259).

Protein phosphorylation is a central regulatory mechanism controlling almost every cellular process, including cell growth, motility, differentiation, and division. The human kinome accounts for nearly 2% of the human genome; as such, it is one of the largest homologous protein superfamilies, consisting of >500 kinases (4). Kinase activity catalyzes the transfer of γ-phosphate of ATP to serine, threonine, tyrosine, or histidine residues on substrate proteins (5, 6, 7). Protein phosphorylation acts as a molecular switch rapidly triggering a shift in protein folding, activity, location, or interaction with other biochemical structures, including proteins, RNA, and DNA. The tight regulation of phosphorylation is essential for "normal" homeostatic control, and mutation or dysregulation of protein kinases or adaptors drives the progression of a wide spectrum of human diseases, including chronic inflammation, cancer, and ischemia (8, 9). Because of their critical roles, kinases are one of the most important targets of precision medicine (10, 11). Consequently, advances in therapeutic approaches are explicitly tied to our understanding of when, where, and how kinases are activated.

### Kinase activation

The general conserved structure of most kinases consists of a bilobal catalytic domain with an N-terminal lobe made of a β-sheet, one α-helix (C-helix), and an α-helical C-terminal lobe (Fig. 2, *A* and *B*) (12). The active site of γ-phosphate transfer is buried in the interface between the two lobes. The ATP-binding pocket is primarily conserved between kinases and surrounded by additional pockets that are less conserved and are often the sites targeted by small-molecule inhibitors (13). The activation of kinases is tightly regulated by interaction with adaptor proteins, as seen for mitotic kinase activation first established for the cyclin-dependent kinase (CDK) family or sequential phosphorylation by kinase signaling pathways. The mitogen-activated protein kinase (MAPK) p38 sub-family is a classic example of the complexity of kinase regulation. All eukaryotes express p38 MAPKs, and their structural and regulatory characteristics are predominantly conserved from yeast to humans. The current models of molecular activation of kinases are still being refined. However, it is widely accepted that the N-terminal lobe facilitates ATP binding. In contrast, the C-lobe mediates effector and substrate binding at the kinase interaction motif (KIM or D-motif), requiring active loop phosphorylation and a conformational change that fully activates the kinase (14, 15). Recent studies challenging this model show that dual phosphorylation of the active loop is required in conjunction with the allosteric modulation induced by substrate binding. Combined, these two forces drive the critical conformational changes in the C- and N-lobes to fully activate the kinase (16, 17, 18).

**Figure 2:**
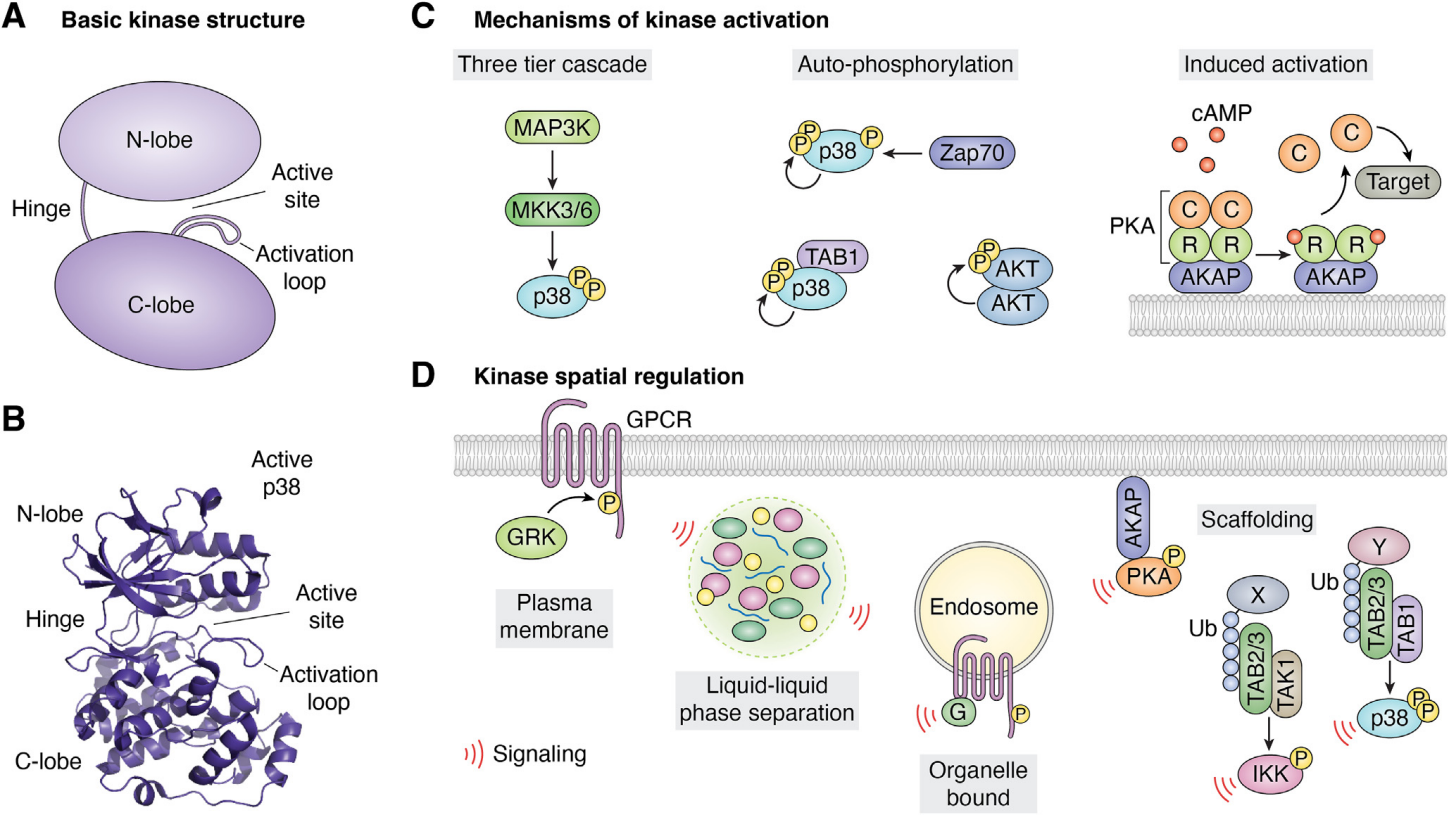
**Modes of Activation and Spatial Regulation of Kinases.***A*, schematic representation of basic kinase structure, including, N-lobe, C-lobe, hinge region, catalytic-loop, and active site. *B*, structural model of TAB1 activated MAPK p38, PDB:4L00, showing the p38 structure. *C*, the basic mechanisms of kinase activation. Divided into three broad categories: i) Three-tier kinase cascades, where MAP3K activates MAP2K, and finally, the MAPK. ii) Autoactivation, where either an adaptor can bind, causing a conformational change and kinase autophosphorylation, or phosphorylation outside of the active loop that causes a conformational change and autophosphorylation, or dimer formation enabling cis- or transphosphorylation. iii) signaling molecular indued activation; in this example, cAMP binds to the regulatory subunit of PKA, triggering activation and release of the catalytic subunit. *D*, spatial regulation of kinases. Plasma membrane: Localization to the plasma membrane initiates GPCR-mediated signaling. G-protein receptor Kinases (GRKs) are recruited to the membrane to phosphorylate GPCRs and induce receptor internalization. LLPS: Compartmentalization of concentrated host factors such as DNA & RNA leads to the formation of LLPS. These membraneless compartments allow for dynamic multivalent interactions within the condensate. The dynamic formation enables fine-tuning of cellular signaling within and downstream signaling out of the LLPS condensate. Organelle: Multiple organelles are spatial hotspots for cellular signaling responses. These include the nucleus, endoplasmic reticulum (ER), mitochondria, and Golgi apparatus (GA). PTM’s of proteins can enhance their membrane binding or translocation to specific organelles and influence their interactions. Scaffolding: Proteins that act as scaffolds help to sequester cytosolic, soluble protein families. This method of compartmentalization initiates local cellular signaling cascades by supporting interactions of adapter proteins, leading to phosphorylation events and downstream signaling. Scaffolding proteins can be localized cytosolically or anchored to organelles such as endosomes, Golgi, ER, and mitochondria. Scaffolding proteins such as ubiquitin and AKAP are the drivers of signaling cascades. AKAP’s act as an anchor for PKA. Similarly, ubiquitin chains drive the recruitment of the adapter protein complex, such as TAB2, leading to the autophosphorylation of p38. cAMP, 3′, 5′-cyclic adenosine monophosphate; LLPS, liquid-liquid phase separation; MAP3K, MAP kinase kinase kinase; PKA, protein kinase A.

Using MAPK p38 as an illustration of the complexity of kinase activation, p38 is classically activated by dual phosphorylation of the active loop (Thr180, Tyr 182) by a MAP2K, which is, in turn, phospho-activated by a MAP kinase kinase kinase. Alternatively, autoactivation of p38 can be induced through either ζ-associated potein of 70 kDA phosphorylation of p38 (Tyr323) or TGFβ–activated kinase one binding protein one (TAB1) binding to p38 (19, 20, 21, 22). In both cases, these events bypass the requirement of MAP2K and cause a conformation change that drives the active loop into the catalytic domain, enabling autophosphorylation (cis-phosphorylation). ζ-Associated potein of 70 kDA-mediated autoactivation occurs only in T-cells and almost exclusively on Thr180, further enabling auto- and trans-phosphorylation between p38α and p38β. Meanwhile, TAB1-mediated activation appears to be broadly applicable to most, if not all, cell types and is associated with autoactivation of p38α (Fig. 2*C*). Autoactivation itself is not exclusively found for p38. Indeed, cis-and trans-autoactivation has been reported for a wide range of kinases. These include pyruvate dehydrogenase kinase1 (PDK1) (23), Aurora Kinase A and B (24, 25), interleukin receptor-associated kinase 4 (IRAK4) (26, 27), and protein kinase D (PKD) (28, 29). However, the mechanism for activation varies and, in many cases, is still controversial. The diverse mechanisms of activation contribute to the differential signal transduction pathways. One way is through allosteric modulation, where cofactor interactions can inhibit or enhance kinase activity and substrate binding. Another is the dynamic sequestration or retention of kinases at specific subcellular locations, as seen by nuclear or cytosolic translocation of activated kinases, which effectively biases their access to specific substrate pools. Spatially defined signaling hotspots enhance the activation of some substrates while blocking or reducing access to others (12, 17, 30).

In turn, hyperactivation of kinases often disrupts the carefully controlled homeostatic balance inside the cell, leading to pathologic responses. Therefore, signal termination is critical to maintain balance. For p38, desensitization requires dephosphorylation of the activation loop, principally through the action of the MAPK phosphatase (MPK)/dual-specificity phosphatases (DUSP) families. Humans have ∼ 250 phosphatases, with many pseudo-phosphatases represented (including TAB1) (31). Each kinase will be regulated by a subset of phosphatases, which tend to be less selective than the kinase activation pathways. Interestingly, activation of most kinases triggers the regulation of negative feedback loops to limit hyperactivation. In our example, p38 can induce DUSP1 and MKP1 to terminate signaling and is important for pro-inflammatory signaling and stress-induced cell death (32, 33, 34). Alternatively, p38 can directly trigger negative feedback by limiting MAP kinase kinase 6 expression (35), or phosphorylating TAB1 after auto-activation, which then blocks TAB1 regulation of the classical MKK3/6 pathway (36).

Taken together, selective activation and desensitization pathways provide a significant array of routes to fine-tune kinase signaling in each context. Therefore, the opportunities for additional regulation are enhanced when you factor in where the kinases are and the locally available adaptor and substrate interactomes.

## Mechanisms of sequestration, protein-protein interactions, and functional compartmentalization

### Adaptors and scaffolding proteins sequester kinases near substrates

Scaffolding proteins help bring order to the soluble pool of proteins in the cytosolic space by regulating specific protein interactions, shaping the local space, and focusing or facilitating specific intracellular signaling responses, including propagation, termination, and localization (37, 38). One well-studied family of scaffolding proteins driving kinase activity are the A-kinase anchoring proteins (AKAP), which direct 3′, 5′-cyclic adenosine monophosphate (cAMP)-dependent protein kinase, or protein kinase A (PKA), a member of the AGC kinase family (39). PKA is a tetrameric holoenzyme composed of dimerized regulatory subunits (RI or RII) bound to two catalytic subunits (Fig. 2*C*). The activation of PKA is facilitated by the release of cAMP-bound regulatory subunits, which allow for diverse downstream signaling responses (40). While cAMP and PKA are cell-diffusible, cAMP has long been suspected of existing in functional gradients (41, 42, 43) and microdomains (44, 45, 46, 47, 48, 49, 50, 51). PKA activity is directed by binding to AKAP proteins to regulate signaling and mediate cross-talk between signaling pathways (52). This is regulated by sequestering PKA within compartments or signalosomes and coupling PKA to substrates and other components of the signaling axis, such as phosphodiesterases (53). AKAPs can positively or negatively regulate signaling by limiting kinase access to substrates. There are over 50 known AKAPs, many of which have alternative splice variants that alter localization (54, 55), such as dAKAP1, which traffics to the endoplasmic reticulum or the mitochondria depending on the presence of an Asp(31) at the N-terminus sequence (56). Other locations AKAPs are trafficked to include the cell membrane, lysosomes, Golgi, nucleus, mitochondria, cytoplasm, and non-membranous signalosomes such as the centrosome and radial spokes (57). Although AKAPs were named for their recruitment of PKA, these proteins act as scaffolds for the assembly of many other signaling pathways in specific compartments such as extracellular signal-related kinase one and two (ERK1/2), hypoxia-inducible factor 1α (HIF-1α), p68 RNA helicase, cyclins, proto-oncogene cellular-sarcoma and Calmodulin, among others (58, 59). AKAPs define the specific access of active C units to selective substrates. Almost all AKAPs have a PKA RII subunits binding domain (RIIBD), a highly conserved amphipathic alpha-helix of 14/18 amino acids that bind to the four-helix D/D domain R-subunit dimers (60, 61, 62, 63). AKAPs localize with bound PKA at subcellular locations *via* protein-lipid or protein-protein interaction (55). AKAPs undergo PTMs, including myristoylation/palmitoylation, to draw PKA toward its substrates and enhance membrane localization. For example, palmitoylation of AKAP150 promotes localization to recycling endosomes and post-synaptic density in neurons, promoting PKA-regulated α-amino-3-hydroxy-5-methyl-4-isoxazolepropionic acid receptor trafficking (64, 65), while Gravin is myristoylated and AKAP18 is myristoylated and palmitoylated to facilitate docking to the plasma membrane (66, 67). Some AKAPs, such as AKAP450, have a pericentrin-AKAP450 centrosomal targeting (PACT) domain, known to interact with Cdc42-interacting protein-4, a cdc42 effector regulating actin dynamics that drives localization to the Golgi and centrosomes (68, 69).

Alternatively, the RTK family can directly act as scaffolding proteins. These single-pass transmembrane receptors have cytoplasmic-facing tyrosine kinase domains that autophosphorylate in trans. The phospho-group facilitates the recruitment of downstream signaling proteins, usually using a Src homology two (SH2) or phosphotyrosine-binding (PTB) domain (70). In addition, signaling is further defined depending on protein polarization to the apical or basolateral membranes in the epithelium, which can drive signaling responses and cancer progression (71, 72). RTK activation also modulates the localization of Phosphatidylinositol 3-kinase (PI3K)-AKT signaling at intracellular membranes. After stimulation, PI3Kα is trafficked along microtubules *via* direct binding of microtubule-associated protein 4 (MAP4), leading to PI3K interaction with receptors at endosomal compartments and contributing to the generation of phosphatidylinositol-3,4,5-trisphosphate (PIP3) and AKT activation (73). PI3K also plays a central role in regulating plasma membrane signaling dynamics. Controlling cellular responses to membrane-bound receptor activation increases PIP3 concentrations and the subsequent activation of protein kinase D and AKT (74, 75, 76). As such, the relative concentrations of lipids in cellular organelles play critical roles in regulating protein association and activation. They also directly impact membrane topology, trafficking, and signaling transduction (77, 78, 79, 80, 81). Although beyond the scope of this review, lipid membrane microdomains or nanodomains of lipids can, in turn, play essential roles in regulating kinase recruitment and activation, including caveolae lipid raft recruitment of PKC (82) and diacylglycerol activation of PKC (83). However, the broader implications of lipid microdomains/nanodomains have been investigated and reviewed extensively (84, 85, 86).

AKT signaling also drives changes to the subcellular localization of other proteins, including SR Protein Kinases (SRPKs), where direct (87) or indirect phosphorylation (88) by AKT drives nuclear translocation. Subcellular localization can also be regulated by scaffold or adaptor proteins. In this example, SRPK's association with heat shock protein (HSP) can act as a cytosolic anchor (89, 90) or facilitator of translocation to the nucleus (88). Alternatively, selective scaffold proteins, JNK interacting proteins one and two (JIP1 and JIP2), bind to form oligomeric complexes that facilitate JNK activation and signal transduction in the cytoplasm near the plasma membrane by aggregating activators of the JNK kinase module (91).

Localization can be more complex, as seen for AMP-activated protein kinase (AMPK, where activity can be localized to the Golgi, endoplasmic reticulum, mitochondria, and lysosome in response to metabolic inhibition or genetic knockdown (92, 93, 94). Lysosomal localization requires AMPK and Liver Kinase B1 (LKB1) to form a complex with AXIN and Late Endosomal/Lysosomal Adaptor, MAPK And mammalian target of rapamycin Activator 1 (LAMTOR1) (95, 96). AMPKα subunits are necessary for cytosolic and nuclear localization and contain sequences recognized by nuclear transport receptors (NTR) (97, 98). However, although a nuclear localization sequence (NLS) in AMPKα2 has been proposed to be necessary for nuclear transport (99) and the NLS is typically recognized by the nuclear translocation importin family of proteins (100), direct evidence for importin interaction with NLS-containing AMPKα2 has not yet been shown (101, 102). There is also evidence that specific AMPK isoforms (α1/α2/β2/γ1) are localized to the outer mitochondrial membrane, and activity only upregulated during more severe metabolic stress, indicating that there may be a hierarchical activation of AMPK at different subcellular locations depending on the severity of stress (103). Scaffolding proteins can also assemble core components critical for tiered kinase activation, as seen for ERK1/2. Kinase suppressor of Ras 1 (KSR1) and isoleucine and glutamine motif-containing guanosine triphosphatase activating protein 1 (IQGAP1) form a complex, where mitogen-activated protein kinase kinase (MEK) bound to IQGAP activates ERK1/2 bound to KSR1 at defined signaling puncta in the cytosol (104).

Lastly, MAPK p38 signaling can be directly modulated by binding to adaptors and scaffolding proteins. The spatial localization can be controlled by binding nuclear importins that induce nuclear translocation (105, 106). Meanwhile, p38 binding to TAB1 spatially restricts the kinase to the cytosol, limiting nuclear translocation and directly biasing or reducing access to nuclear substrates (107, 108). Conversely, the interaction with scaffolding proteins such as discs-large homolog 1, protein osmosensing scaffold for MAP3K/MEKK3, or JIP4 can localize p38 to specific cell areas while also orchestrating intersection with other signaling pathways by tethering p38 to alternate complexes (109, 110, 111).

### Spatially biased G-protein coupled receptor (GPCR)-Dependent signaling

GPCRs are typically associated with activating heterotrimeric G proteins following agonist stimulation at the plasma membrane, resulting in subsequent activation of effectors and secondary messengers. Recent studies have established that intracellular GPCRs can orchestrate spatially restricted G-protein signaling at sub-cellular compartments. These include the nucleus, endoplasmic reticulum, mitochondrion, Golgi, and the early endosome, and have been reviewed (112). Additionally, G protein signaling drives the activation of secondary messengers that initiate the regulation and activation of kinases. β-arrestins are multivalent adaptors that bind GPCRs and scaffold essential signaling complexes such as the c-RAF-MEK1-ERK1/2 signaling cascade (113). Prior models postulated that GPCR-induced plasma membrane recruitment and activation of ERK1/2 (114, 115). However, recent work challenges this hypothesis by showing that GPCR-stimulated MAPK signaling can also occur independently from β–arrestins (116, 117) and that ligand-induced GPCR endocytosis displays distinct subcellular ERK1/2 signaling dynamics (118, 119). Paired with the known subcellular ERK activity (120), these studies led investigators to explore subcellular pockets of ERK activity after endocytosis of β2-adrenergic receptor (β2-AR), discovering that GPCR activity does not result in ERK1/2 activation at the plasma membrane but is localized to the endosome (121). This mechanism was further explored and found to be mediated by a signaling axis involving endosomal-localized active G_αS_-Raf-MEK, resulting in endosomal activation of ERK (121). The investigators noted that active ERK was then trafficked to the nucleus, affecting the activation of downstream transcription factors, suggesting that this was facilitated by the proximity of the nucleus to the endosome (121).

In addition to AKAP-directed PKA localization, it has been shown that the location of GPCR activation within the cell can also drive compartmentalized PKA activity. β2-AR signaling at the endosome drives active PKA localization to the nucleus in a PDE-dependent manner; however, disrupted endosomal GPCR activation or endosome location perturbs PKA function in the nucleus (122). These findings illustrate that compartmentalized signaling can protect kinase activity by shortening the distance the kinase travels in a cellular environment that's "hostile" to active kinases.

### Ubiquitin as a signaling scaffold and spatially restricted kinase activation

Figure 1 summarizes how PTMs can impact cellular functions. Polyubiquitination classically regulates protein trafficking and degradation but can also scaffold the formation of spatially restricted kinase signaling complexes (123). Tumor necrosis factor (TNF)-mediated pro-survival signaling involves the IKK (IkB kinase) and TAK1/TAB2/3 (transforming growth factor beta-activated kinase 1, TAK1-binding proteins two/3), which are recruited in a ubiquitination-dependent manner. Specifically, K63 polyubiquitin chains recruit TAB2/3 *via* their carboxy-terminal zinc-finger (ZnF) domains that then recruit TAK1. These resulting signaling complexes are important for activating NF-kB and MAPK c-Jun N-terminal kinase (JNK) pathways (124, 125).

Correspondingly, K63 polyubiquitin of the C-terminal tail of activated GPCRs facilitates the scaffolding or recruitment of TAB2/3 to endosomes. This initiates the formation of a TAB2-TAB1-p38 complex, resulting in the autophosphorylation of p38 at the endosome (126, 127). Notably, GPCR-mediated p38 activation is focused away from the nucleus (107), contrary to osmotic-stress-induced p38 activation, which is driven to the nucleus (107). This finding provides some of the first evidence of a mechanism of p38 sequestration away from the nucleus, biasing p38 toward cytosolic substrate pools rather than nuclear transcription factors (128). This ubiquitin-dependent sequestration mechanism differs from the recently uncovered MAPK ERK1/2 endosomal activation, but suggests a conserved strategy to spatially bias GPCR-induced MAPK activity (Fig. 2*D*).

### Liquid-liquid phase separation-dependent sequestration of kinase activity

The eukaryotic cellular organization also extends to membrane-less compartments that transiently occur or form in stress responses known as molecular condensates or liquid-liquid phase separation (LLPS). Broadly speaking, biomolecular condensates comprise biomolecules with structural features that allow multivalent interactions between proteins, RNA, and DNA, inducing LLPS formation. The physical properties of these condensates exhibit liquid-like behavior and form as the result of phase transitions when the molecular components of the condensates coalesce to form LLPS. Phospholipid metabolism can also contribute to the formation of phase-separated condensates (129). LLPS enable a dynamic localized layer of regulation that can be turned on and off by their formation and dissolution. Proteins capable of forming these condensates also typically have intrinsically disordered regions (IDR) with low amino acid complexity but can also be highly structured proteins that oligomerize into higher structures (130). It is becoming increasingly clear that LLPS condensates play important roles in kinase signal transduction (131) and are increasingly a focus when exploring spatial signaling dynamics; see (Fig. 2*D*) (Table 1).

**Table 1:** Kinases associated with LLPS condensates

Kinase	Type of condensate	Mechanism of kinase-condensate localization	Source (DOI)
Plk4	Centriole assembly	Phosphorylation-dependent intrinsic phase separation	https://doi.org/10.1038/s41467-019-09847-x
PKA	Condensates with cAMP	RIα-mediated intrinsic tendency	https://doi.org/10.1016/j.cell.2020.07.043
MARK2	Condensates with Tau protein	Possibly recruited by substrate TAU	https://doi.org/10.15252/embj.201798049
FAK	Integrin-mediated adhesion complexes	Intrinsic scaffolding with p130Cas	https://doi.org/10.7554/eLife.72588
TBK1	polyubiquitin condensates	Recruited by adaptor proteins	https://doi.org/10.1038/s41598-021-92408-4
ULK1	polyubiquitin condensates	Recruited by adaptor proteins	https://doi.org/10.1016/j.molcel.2019.01.035, https://doi.org/10.15252/embr.202051136, https://doi.org/10.1016/j.molcel.2019.02.010, https://doi.org/10.1042/EBC20170021
TNK1	polyubiquitin condensates	Kinase-intrinsic ubiquitin-binding domain	https://doi.org/10.1038/s41467-021-25622-3
RTKs	RTK-mediated LLPS	Intrinsic phase separation tendency	https://doi.org/10.1016/j.tcb.2023.09.002
S6K1/2	Stress granules	Mechanism unclear, possible interaction with adaptor proteins	https://doi.org/10.1038/s41418-018-0076-9
PKC	Stress granules	P-body associated scaffold proteins G3BP2	https://doi.org/10.1534/genetics.114.172031, https://doi.org/10.1371/journal.pone.0035820
PKR	Stress granules	P-body associated scaffold proteins G3BP1	https://doi.org/10.1128/jvi.02791-14
DYRK3	Stress granules	N-terminus dependent localization to stress granules and intrinisic kinase activity	https://doi.org/10.1016/j.cell.2013.01.033
CK2	Stress granules	P-body associated scaffold proteins G3BP1	https://doi.org/10.1128/MCB.00596-16
Sky1	Stress granules	Intrinsic phase separation tendency	https://doi.org/10.1038/s41467-019-11550-w
Syk	Stress granules	Phosphorylation-dependent recruitment by Grb7	https://doi.org/10.1074/jbc.M115.642900
MAPK p38	Stress granules/viral inclusion bodies/anti-mycobacterial immune LLPS	Recruitment by substrate, viral-mediated sequestration	https://doi.org/10.26508/lsa.201800257, https://doi.org/10.1128/jvi.02263-12
Lck/ZAP70	T-cell signaling phase-separated droplets	Recruited by TCR	http://doi.org/10.1126/science.aad9964

Abbreviations: CK2, casein kinase 2; DRK3, dual specificity tyrosine-phosphorylation-regulated kinase 3; FAK, focal adhesion kinase; S6K1/2, ribosomal protein S6 kinase beta-1/2; SYK, spleen tyrosine kinase; TBK1, F family member associated NFKB activator (TANK)-binding kinase 1; ULK1, unc-51-like kinase 1; ZAP70, ζ-associated potein of 70 kDA.

In addition to its roles as a membrane scaffolding protein, described above, polyubiquitin chains also show properties of liquid-liquid phase separation. Specifically, after interleukin-1 (IL1) or TNFα stimulation, the NF-kB essential modulator (NEMO) regulatory subunit of IkB kinase (IKK) is known to phosphorylate the inhibitory IkBs to facilitate activation of IKK, which it accomplishes *via* binding to polyubiquitin chains (polyUb) (132). Additionally, linear ubiquitin chain assembly complex (LUBAC)-catalyzed M1-and Lys63-linked linear polyUb binding to NEMO, resulting in the formation of liquid-like droplets. Phase-separation formed modules of IKK activation, and mutations associated with the loss of this phase-separation are associated with immunodeficiency (133, 134).

As discussed above, AKAP directs PKA function to selective organelles. Recent studies have revealed that a non-canonical higher-level organization also occurs, where the type I regulatory subunit of cAMP-dependent PKA, RIα, undergoes phase separation in response to GPCR-induced cAMP signaling. RIα LLPS biomolecular condensates enable compartmentalization of cAMP but also restrict the movement of catalytic subunit (PKA-C), concentrating activity in LLPS. The D/D and cyclic nucleotide-binding-A domains are both required for LLPS formation, which act as cAMP "sponges" that increase localized microdomains of cAMP that provide protection from cAMP-hydrolyzing phosphodiesterases (PDEs) activity (135, 136).

### Viral-associated LLPS

Viruses coerce host cellular machinery to enhance their replication and manipulate immune responses. A growing body of evidence suggests that DNA and RNA viruses can compartmentalize viral and host factors to enhance their replication (137) and manipulate immune responses (138). A wide variety of terminologies are used for these viral events, including LLPS (139), bimolecular condensates (137, 140), processing bodies (138, 141, 142, 143, 144), cytoplasmic membrane-less organelles (145, 146, 147), stress granules (SG) (148, 149, 150), cytoplasmic Virion Assembly Compartments (cVACs) (151, 152, 153), or nucleocapsid assembly sites/inclusion bodies (IB) (137, 154, 155). Condensates are induced through multiple factors, including cellular pH, rapid increase in host cell nucleic acid concentrations, binding of heat shock proteins (HSP70), and activation of host kinases, including double-stranded RNA-dependent protein kinase (PRK) (156, 157).

DNA viruses (such as herpesvirus (153)) utilize nuclear inclusion bodies to transcribe the viral genome and cVACs for virion assembly and maturation (139). Herpes Simplex Virus 1 (HSV-1) progeny are assembled in membraneless viral replication compartments within the nucleus (158), demonstrating the properties of LLPS (139, 159). RNA viruses such as Respiratory Syncytial Virus (RSV) (160), Human Metapneumovirus (HMPV) (161), and Human Immunodeficiency Virus (HIV-1) (162) also utilize bimolecular condensates. During RSV infection, the NFκB protein, p65, translocates to inclusion bodies containing viral components (160), enhancing proinflammatory signaling and the host immune response (163). Conversely, RSV sequesters host proteins *O*-GlcNAc transferase (OGT) and phosphorylated p38 (164). Retaining p38 to the condensate impairs p38-induced MAPK2 activity, limiting NFκB signaling (156). Most viruses intersect with the NFκB pathway. Therefore, it is tempting to postulate that they all likely directly impact host kinase localizations and activities through PRK activation and Ub-mediated LLPS formation.

## Tools developed to study spatial signaling

**Figure 3:**
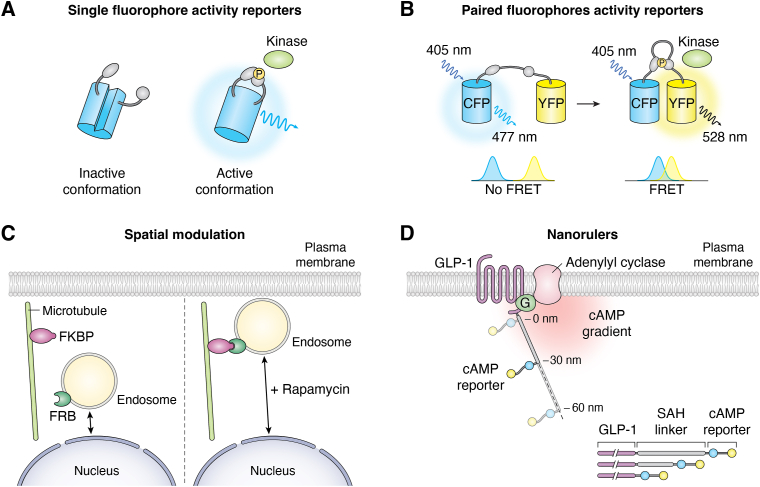
**Kinase Activity Imaging Tools with Subcellular Resolution.***A*, single fluorophore activity reporters. This type of reporter utilizes a circularly permuted fluorophore tethered to a sensing unit composed of a kinase-specific substrate and phosphoamino acid binding domain (PAABD). Phosphorylation of the substrate results in a conformational change in the fluorescent protein, altering fluorescence readout. *B*, paired fluorophore activity reporters. This type of reporter utilizes tethered fluorophores joined by a linker peptide containing a kinase-specific substrate and PAABD. Phosphorylation of the substrate results in a conformation change of the peptide, bringing the fluorophores into close physical proximity, allowing for the transfer of fluorescence energy from the donor (CFP) fluorophore to the acceptor (YFP) and detection of kinase-mediated FRET activity *via* acceptor emission. *C*, spatial modulation. This rapamycin inducible system modulates endosomal localization using early-endosomal antigen 1 (EEA1)-tethered FK506-binding protein-rapamycin-binding (FRB) fused to GFP alongside a plus-end-directed kinesin motor Kif1a fused to a FK506-binding protein (FKBP) and tandem dTomato, mediating microtubule and kinesin motor directed migration of endosomes away from the nucleus. *D*, nanorulers. This system utilizes fluorescence biosensors such as Epac1-camps separated by rigid single-alpha-helical (SAH) domain linkers of set size to measure cAMP gradients at different locations within the cell.

To study the critical transient biochemical events that regulate subcellular signal transduction, a variety of tools have been developed that allow real-time reporting of compartmentalized events or augmentation of trafficking machinery. Many of these tools are genetically encoded optical biosensors designed to use fluorescence as a readout for a biochemical event, such as kinase phosphorylation. These biosensors have a kinase-responsive component that induces changes in conformation, altering fluorescence that can be detected by high-resolution microscopy. Fluorescence Resonance Energy Transfer (FRET) is a proximity-dependent energy transfer from a donor molecule to an acceptor molecule *via* dipole-dipole interactions. This electrodynamic phenomenon is frequently utilized in biosensor designs because for FRET to occur, the donor molecule must be within close physical proximity to the acceptor molecule, directly enabling the development of sensitive, specific biosensor reporters (Fig. 3). The field of genetically encoded biosensors is extensive and has been excellently reviewed elsewhere (165). Here we will focus on some of the ways that fluorescence and FRET-based biochemical tools allow us to study subcellular kinase signaling.

### Single and tethered fluorophore activity reporters

Single fluorophore biosensors are straightforward methods of detecting biochemical activity through the integration of a molecular switch on the beta-barrel of a fluorophore that alters fluorescence emission based on biosensor conformation (Fig. 3*A*). A strength of these reporters is that they can be multiplexed in a single cell to measure multiple cellular processes simultaneously, for example, the detection of PKA, PKC, and ERK signaling (166). Single-fluorophore kinase activity reporters can also elucidate compartmentalized kinase signaling through fluorescence changes rather than translocation. An example is the excitation-ratiometric AMPK activity reporter (ExRai AMPKAR), which detects endogenous AMPK in this manner. The biosensor was directed to measure cytoplasmic, lysosomal, mitochondrial, and nuclear AMPK (93).

Kinase translocation reporters (KTRs) are used to study kinase activity *via* their design of a fluorophore fused to a kinase substrate and a bipartite nuclear localization signal (bNLS) and nuclear export signal (NES). The sensor's phosphorylation imparts a negative charge that modulates nuclear import/export. KTRs facilitate rapid kinase activity measurement as a cytoplasmic to nuclear ratio of fluorescence and can be multiplexed to track multiple kinases in a single cell when using spectrally separated fluorophores but are nonspecific to kinase distribution within the cell (167).

Tethered fluorophore biosensors utilize FRET to report kinase activity. This reporter class was first used to detect Ca2+ signaling using a calcium-sensing calmodulin linked to a peptide derived from the myosin light chain, between a blue fluorescent protein and YFP (168). Sensitivity was enhanced by exchanging the fluorophores for enhanced YFP (EYFP) and enhanced cyan fluorescent protein (ECFP) (169), the general design of which became the basis for subsequent biosensors, including kinase activity reporters. Alternatively, “bait” substrate amino acid sequence specific for a kinase of interest can be linked to a flexible peptide containing a phosphoamino acid binding domain linking together a pair of FRET fluorophores. Phosphorylation of the bait substrate by the target kinase causes the phospho-recognition domain to bind to it, facilitating a conformational change in the peptide and drawing the fluorophores into close proximity where FRET can occur (Fig. 3*B*). Tethered-fluorophore kinase activity reporters have been designed and utilized for many kinases, from PKA, PKC, PKB/Akt, MTORC1, aurora kinases, ERK1/2, and p38 (32, 94, 107, 170, 171, 172, 173, 174, 175, 176, 177). These activity reporters have been further refined as improved fluorophores are designed and implemented. Critically, the growing library of kinase reporters can be further modified with subcellular localization motifs that direct the activity reporters to other subcellular locations such as lipid rafts, the plasma membrane, endosome, mitochondria, or nucleus (107, 121, 178, 179). An online database listing many of these biosensors can be found at https://biosensordb.ucsd.edu/. Spatial targeting of these biosensors enables detailed studies of how biomolecular factors control kinase associated with organelles within each cell, revealing key insights into spatiotemporal dynamics of kinase activity and how they drive disease-inducing signaling transduction (107, 121, 136, 177).

### Tools to study subcellular kinase activity

Aside from kinase activity reporters, investigators have developed novel tools to delineate and better understand kinase substrate availability and the subcellular context of signaling. Using a genetically encoded system of rapamycin-induced endosomal redistribution, fusion proteins anchored in the endosome and plasma membrane can be manipulated to direct the endosome to the cell periphery (Fig. 3*C*). Using this model, the investigators uncovered the functional roles of PDE-mediated cAMP hydrolysis and location-specific PKA activity (122). To better understand compartmentalized PKA signaling and cAMP-degrading phosphodiesterase (PDE) activity, investigators developed FRET nanorulers to measure nanometer-size cAMP gradients in intact cells. These nanorulers consist of a FRET-based cAMP reporter (Epac1-camps) (180) and a PDE, separated by single alpha-helices (SAH) of set nanometer length (Fig. 3*D*). These rigid ER/K helices allow for precise measurement of the distance between active PDEs and cAMP biosensors, representing molecular rulers to measure the size of low cAMP pockets generated by PDE activity. These were subsequently used to determine that cAMP buffering (135) and local PDE activity can “protect” cAMP effectors from activation until cAMP concentrations are sufficiently high to overcome buffering and initiate signaling responses (181).

## Roles of kinase in cellular compartments and their impact on physiology

As our understanding of the mechanism driving the spatial compartmentalization of kinases and the tools to detect the spatial bias are refined, so is our understanding of the physiological and pathophysiological impact. Below, we discuss some of the latest studies describing compartmentalized signaling utilized in physiology and how mutations that disrupt or alter kinase localization can result in disease.

Since their discovery as modulators of PKA and other kinase activity, AKAPs have long been considered attractive avenues for therapeutic intervention, as AKAPs play key roles in cancer, ischemic stroke, and cardiovascular pathologies such as cardiac hypertrophy, heart failure, and hypertension (57, 182, 183), among others. These diseases generally arise from mutations or changes in the expression of the AKAP proteins, causing them to lose or alter function, leading to mislocalized or loss of effective PKA signaling, substrate sequestration, or dysregulated increases in signaling responses. For example, AKAP-Lbc is needed for both cardiac development as it localizes to the cytoskeleton and scaffolds with many kinase binding partners, but is also responsible for the development of pathological myocyte hypertrophy when upregulated, due to an increase in MEF2-mediated transcriptional reprogramming (184, 185, 186). Conversely, loss of AKAP-Lbc *via* miR-629-5p results in malignant prostate cancer (187). Mutations in PKAc itself, however, can also alter localization, leading to disease. An example of these is the PKAc^W196G^ mutation that increases kinase affinity for RI-type regulatory subunits and their associated AKAPs, resulting in Cushing's syndrome (188).

AMPK is an essential energy sensor and master regulator of cellular metabolism that is subcellularly localized to monitor and react to cellular energy states and response to stress (189). Zong *et al*. investigated how signaling cues regulate differential subcellular AMPK activation (103). They discovered that early-stage glucose starvation leads to lysosomal AMPK activation *via* the aldolase-v-ATPase-Regulator-AXIN-LKB1 complex, leading to phosphorylation of downstream substrates. However, moderate starvation increased AMP levels corresponding with lysosomal and cytosolic AMPK activation, whereas severe energy stress resulted in high levels of AMPK activity in the cytosol, lysosome, and mitochondria. In addition to this work, it was also shown that phosphorylation of AMPKα also drives the kinase to the nucleus in response to stimulation by leptin, a hormone involved in energy regulation (99). These results indicate a novel mechanism for functional spatial kinase distribution as a sensor of metabolic stress response (103).

### Differential signaling and pathological outcomes by GPCR-activated kinases

Active GPCRs facilitate the recruitment of G-protein-coupled receptor kinases (GRK)s to the plasma membrane, where they bind to and phosphorylate the GPCR. Phospho-patches then recruit arrestins, which can initiate desensitization, receptor internalization, and internalized signaling (190, 191). GRKs have additional roles; for example, GRK5 possesses an NLS motif enabling nuclear translocation in myocytes to facilitate maladaptive growth and fibrosis in a calcium/calmodulin (CaM) dependent manner (192). Alternatively, GRK2 in cardiac tissue is shown to translocate to the mitochondria during myocardial ischemia and oxidative stress in an ERK and HSP90-dependent manner, driving alteration of mitochondrial substrate utilization (193, 194, 195, 196). GRK2 mitochondrial translocation also occurs in response to LPS, leading to increased mt-DNA transcription, reduced ROS, and cytokine expression (59) during inflammatory disease. Much remains unknown about kinase substrate pools in the mitochondrial compartment in a disease context, and further research is needed.

The original models for GPCR desensitization required GRK phosphorylation and GPCR internalization to the endosome principally to terminate signal transduction. However, it’s clear that the signaling landscape of internalized GPCRs is diverse and facilitates subcellular compartmentalized kinase signaling. Ligand-stimulated, internalized β2-AR facilitates Gαs-mediated ERK activation at the endosome, regulating cell proliferation through nuclear-transported ERK and upregulation of substrates like nuclear cellular myelocytomatosish. These findings identify a functional role for internalized signaling and could be linked to cancer progression (121).

Conversely, stimulation triggers spatially distinct pools of MAPK p38 activation. P38 has over 100 substrates and is responsible for physiological cell development, growth, injury responses and dysregulated pathological inflammation (197). Much research has gone into deciphering the context of p38 signaling in health and disease to identify novel therapeutic strategies. Recent studies argue that adaptor binding and spatial distribution is central to the physiological and pathological outcomes. The clear nuclear-cytosolic distribution of p38 differentially controls access to subsets of substrates (128). It remains a matter of intense research to define how the conserved dually phosphorylated p38 can be switched to pathological outcomes in both the classical and atypical p38 pathways and what differentiates them. One possibility is that spatially selective substrates/adaptors can allosterically modulate the binding potential for secondary targets of p38 (17, 18, 37). One example is the spatiotemporal signaling bias exerted by TAB1-dependent p38 autophosphorylation (107). FRET biosensors revealed that atypical p38 is differentially localized, displaying enhanced cytosolic activity and restricted nuclear access (107), disrupting access to key nuclear targets and increasing access to cytosolic targets. The direct TAB1-p38α interaction drives atypical p38 signaling-dependent vascular inflammation and edema (126, 198, 199), cardiac ischemic damage (19, 20, 200), amyloidosis (201), dermal inflammation (202), and viral replication (203). Further studies are required to determine the molecular mechanisms of signal transduction in each case and whether a conserved spatial bias is critical to driving these pathologies.

### Kinases involved with liquid-liquid phase separation drive physiological processes and disease

Ribonucleoproteins (RNPs) and other LLPS-driven cellular mechanisms are increasingly studied for their roles in physiology and disease (204). We’ve discussed how LLPS mechanisms sequester kinases, but kinases also play key roles in the formation, function, and maintenance of LLPS (Table.1). The centromere, essential for cell division, contains disordered histone tails that cause it to exhibit macromolecule phase separation and interact with nucleosomes. Kinase activity within the inner centromere is driven by LLPS activity (205), and in turn, both LLPS and phosphorylation *via* Aurora kinases drive effective centromere function during mitosis (206). Similarly, viral infections rapidly increase the amount of cytosolic nucleotides, whose negative charge and multivalency promote RNA-protein and protein-protein interactions, inducing phase separation into LLPS. The condensed nucleotides drive local nucleotide concentrations higher, activating double-stranded RNA-dependent protein kinase (PKR) and PKR-like endoplasmic reticulum kinase (PERK) at the ER to initiate the integrated stress response (ISR) and formation of stress granules and the antiviral responses (207). However, multiple viruses also manipulate the PKR responses, to enhance viral production, as seen in the case of the Hepatitis C Virus (HCV) (208). Alternatively, other viruses co-opt stress granule (SG) protein G3BP1 to inhibit PKR and prevent ISR (209). Thus, host kinases can be instrumental in forming and controlling pro- and antiviral SGs (210).

Processing bodies (P-bodies) are formed transiently in the cell from untranslated mRNA associated with decay machinery and other protein interactors in response to stimuli. Protein kinase recruitment to these particles initiates signaling responses, sequestering kinases away from other substrates during cellular responses and stabilizing key proteins co-localized to the granules. PKA is a key regulator of P-body assembly and is hypothesized to regulate the formation of larger P-body aggregates by phosphorylation of Pat1, a key constituent of p-bodies (211). NF-kB is also involved in the formation of p-bodies. IkB complex IKK controls p-body formation through binding and phosphorylation of enhancer of decapping 4 (EDC4), leading to recruitment of mRNA-decapping enzyme complex subunit 1a and two (DCP1a and DCP2). In the absence of stimulus, the IKK-EDC4 complex promotes the degradation of pro-inflammatory mRNAs, indicating diverse roles of kinases within p-bodies (212).

Subcellular kinase activity can dynamically control LLPS condensates (213), where phase separation is both positively and negatively affected by phosphorylation, controlling both aromatic-cationic and aromatic-aromatic interactions (214). For example, DNA-dependent protein kinase (DNA-PK) phosphorylates the neurodegeneration-linked RBP Fused in Sarcoma/Translocated in LipoSarcoma (FUS) protein (215, 216, 217), interrupting phase separation and reducing binding affinity to low complexity domain hydrogels (218, 219, 220, 221). Conversely, Tau protein binds RNA and induces fibrilization, which forms insoluble neurofibrillary tangles during Alzheimer’s disease (222, 223, 224). Serine phosphorylation of the microtubule-binding region of Tau protein by microtubule-affinity regulating kinase two (MARK2) kinase promotes LLPS of Tau protein (225) and possibly contributes to the pathology. Further studies are needed to determine if targeting kinase activity for LLPS modulation could perturb disease progression or offer novel therapeutic avenues.

## Conclusions and ongoing challenges

It is becoming increasingly clear that current therapeutic strategies fall short of certain valuable kinase targets. Defining the mechanisms that control the spatial and temporal control of kinases and their impact on subcellular compartments represent a relatively untapped focus of therapeutic development. As described above, there is a growing acceptance that spatially distinct signaling defines the pathological bias in kinase signal transduction.

However, there are many ongoing challenges in studying spatial kinase activity. For one, it is difficult to identify substrate pools specific to subcellular localization and directly correlate endogenous, localized kinase activity with their substrates. Additionally, the spatial regulation of phosphatase, proteases, and specific localized substrate access requires the development of additional tools. One of the benefits of utilizing biosensors to detect kinase activity is it allows the study of endogenous protein kinase without their overexpression and the associated potential for off-target effects. However, exogenous expression of the biosensor itself could also trigger secondary effects by introducing foreign proteins to a balanced system or taking up essential “space’ on membranes. This sterically blocks access for endogenous proteins, nudging signaling out of its endogenous temporal or spatial state. The “bait” kinase reporters are typically derived from native proteins and therefore could outcompete endogenous kinase substrates.

Additionally, subcellular biosensor targeting can lead to overexpression or saturation at the location of interest, possibly leading to false reports of FRET activity as tethered fluorophores from different individual molecules emit FRET from interactions in *trans*. As brighter fluorophores are developed, expression can be minimized to avoid saturation. Additionally, these studies are typically performed *in vitro* and imaging *in vivo* remains challenging due to low penetrance of fluorescence through complex tissues. This is being addressed through alternative imaging strategies such as multi-photon excitation systems (226, 227, 228) and fluorophore pairs that excite and emit near the infrared spectrum (229).

Spatial signaling offers an opportunity to personalize kinase inhibitors to a specific subset of pathological kinase responses. For example, MAPK p38α is an extensively studied kinase, and several recent reviews have covered the kinase’s roles and therapeutic potential for cancer (230, 231), arthritis (232), viral regulation (233, 234), and other inflammatory disorders (235). However, clinical trials for small molecule drug inhibitors of p38α have largely failed in the clinic. For p38α, this research is increasingly focused on MK2 (232, 236, 237). With greater knowledge of the mechanisms of kinase sequestration and access to substrate pools, we suggest there is the potential to develop strategies that block subsets of kinase activity associated with pathological outcomes. A molecular mechanism driven by GPCR polyubiquitination and recruitment of the active kinase to the endosome *via* TAB1 binding and autophosphorylation has recently been discovered (126, 198). However, therapeutic targeting of spatially restricted MAPK has remained underexplored.

Conversely, spatially targeted polo-like kinase 1 or AURKA kinase (AurA) inhibitors (Local kinase inhibitors, LoKI) can selectively block kinase activity, disrupting microtubule-kinetochore functions (238). Establishing a valuable tool to study spatial signaling and setting a precedent for the future development of other LoKI. However, clinically viable spatially targeted LoKI have yet to be developed.

An alternative emerging therapeutic strategy in perturbing kinase activation is the modulation of LLPS condensates. This field is rapidly growing as we learn how kinases are recruited to condensates, which are similarly essential for kinase function (131). The protein-protein biophysical interactions between IDRs that induce LLPS play key roles in diseases such as Alzheimer’s, ALS, Huntington’s disease, and other neurological conditions. Many groups are now investigating the interruption of LLPS as a therapeutic strategy (239). LLPS could be controlled by targeting kinase activity responsible for nucleation of LLPS condensates or through selective targeting of subsets of kinases trafficked to condensates *via* co-localization of small molecule inhibitors to condensates.

The development of selective and efficacious therapeutics that target the adaptors or scaffolds for subcellular location may be challenging as, in many cases, the molecular drivers of kinase activity are conserved. However, spatially targeted inhibition represents an exciting, relatively untapped approach to selectively block pathological signaling and potentially reduce some of the current strategies' toxicity or off-target impacts.

In summary, a wide range of finely tuned processes regulate the cell's kinase activity. A greater understanding of the mechanisms that drive spatial and temporal kinase activity and the development of advanced tools to visualize and dissect their dynamic activation will likely provide essential insight into how dysregulated kinase signaling drives disease. Critically, these studies are essential to inform the future development of spatially targeted therapeutics.

## Data availability

Data supporting the findings of this study are available from the corresponding author on reasonable request.

## Conflict of interest

The authors declare that they have no conflicts of interest with the contents of this article.
